# The Role of Format Familiarity and Word Frequency in Chinese Reading

**DOI:** 10.16910/jemr.16.4.5

**Published:** 2023-11-29

**Authors:** Ming Jing Chen, Jia Mei Lu

**Affiliations:** Shanghai Normal University, Faculty of Psychology, China

**Keywords:** Eye movement, Chinese reading, word segmentation, vocabulary recognition, E-Z reader model

## Abstract

For Chinese readers, reading from left to right is the norm, while reading from right to left is unfamiliar. This study comprises two experiments investigating how format familiarity and word frequency affect reading by Chinese people. Experiment 1 examines the roles of format familiarity (reading from left to right is the familiar Chinese format, and reading from right to left is the unfamiliar Chinese format) and word frequency in vocabulary recognition. Forty students read the same Chinese sentences from left to right and from right to left. Target words were divided into high and low frequency words. In Experiment 2, participants engaged in right-to-left reading training for 10 days to test whether their right-to-left reading performance could be improved. The study yields several main findings. First, format familiarity affects vocabulary recognition. Participants reading from left to right had shorter fixation times, higher skipping rates, and viewing positions closer to word center.. Second, word frequency affects vocabulary recognition in Chinese reading. Third, right-to-left reading training could improve reading performance. In the early indexes, the interaction effect of format familiarity and word frequency was significant. There was also a significant word-frequency effect from left to right but not from right to left. Therefore, word segmentation and vocabulary recognition may be sequential in Chinese reading.

## Introduction

Chinese is a typical ideographic language containing no spaces, thus differing from alphabetic languages such as English (23). The basic unit of reading processing, whether in Chinese or English, is the word (3; 6; 7). For Chinese readers, the primary task while reading is word recognition (7; 8; 10; 16; 18; 37). The reader needs to segment the word from the text and recognize the whole word (10; 13; 17; 25; 27). Scholars have proposed that word segmentation and vocabulary recognition are completed simultaneously by Chinese readers (13). Whether there is an independent word segmentation process in Chinese is the point of contention between the SWFIT model and the E-Z Reader model (27). SWFIT model proposed word segmentation and vocabulary recognition are parallel processing; while the E-Z Reader model proposed word segmentation and vocabulary recognition are processed sequentially in Chinese reading. This debate is essentially over whether Chinese readers use sequential or parallel processing (9; 14). A recent study revealed a trade-off between inter-word spaces as clues of word segmentation and format unfamiliarity (7). If word segmentation and word recognition are indistinguishable in Chinese reading, then format familiarity will also affect the word recognition stage. In this study, format familiarity refers to the reading direction. Whereas ancient Chinese was read from top to bottom and from right to left, modern Chinese is read from left to right (40). Right to left reading is the norm in Hebrew and Uyghur but unfamiliar to Chinese readers. The purpose of this study is to investigate the processing mechanism of word segmentation and recognition in Chinese reading.

The word frequency effect usually manifests in the vocabulary-recognition stage: it takes longer to process low-frequency (LF) words than high-frequency (HF) words (e.g, 21; 9). Moreover, the skipping probability of the HF words is higher than for LF words (26; 29; 28; 38; 41). Some studies manipulate word frequency to examine the mechanism and characteristics of lexical processing (21; 22; 32). Using survival analysis, Ma (21) found that the word-frequency effect was delayed by 21 ms for unspaced (vs. spaced) text. There was no interaction between word frequency and inter-word spaces, thus excluding the familiarity hypothesis. Ma proposed that inter-word spaces reduce horizontal masking and promote word segmentation. This hypothesis needs to be tested. Another possibility is that inter-word spaces facilitate lexical processing. If so, then this facilitation is likely offset by format unfamiliarity, since inter-word spaces are not the form for Chinese text, which was caused by the lack of reading experience. This trade-off may explain the absence of an interaction between inter-word spaces and word frequency, which Chen et al., (7) also reported. Our study further investigates this assumption. This assumption is whether the trade-off effect reduce the word-frequency effect and delay 21 ms on unfamiliar format.

Our study focuses on a different aspect of format familiarity by manipulating the reading direction, employing both from left to right (the norm for modern Chinese) to right to left (the norm for Uyghur, Hebrew, and ancient Chinese) in two experiments (3). Experiment 1 investigates the individual and interactive effects on reading performance of format familiarity (left to right [familiar] vs. right to left [unfamiliar]) and word frequency (HF vs. LF). If there is an interaction between format familiarity and word frequency, this would indicate that format familiarity can modulate the word-frequency effect on vocabulary recognition. Conversely, no interaction would indicate no modulation. Focusing on eye movement indicators, if there is an interaction between format familiarity and word frequency, it would be valuable to investigate whether this effect differs across processing stages. Accordingly, Experiment 2 involved training participants over 10 days in reading from right to left. Two conditions (LF–right to left, HF–right to left) in Experiment 1 were used as baseline levels, and the same group of participants was used to control for Chinese reading levels. Then, training in reading from right to left could improve readers’ right-to-left reading experience. Experiment 2 compared the results before and after training to investigate whether the reading experience would modulate the word-frequency effect. If this effect is bigger or appears earlier, that would indicate a significant interaction between training and word frequency. A similar interaction in Experiment 2 to that in Experiment 1 would indicate that the role of format familiarity in the word-frequency effect is somewhat explained by reading experience. However, there may be other influencing factors, such as the characteristics of Chinese characters on a visual level. The results in Experiment 1 and 2 provide empirical evidence supporting the Chinese E-Z Reader model or SWIFT model.

## Experiment 1 Methods

The purpose of Experiment 1 is to investigate the role of format familiarity and word frequency in Chinese vocabulary recognition.

### Participants

Forty undergraduate students participated in Experiment 1, which contained 31 females and 9 males (mean age 20.50 ± 1.63 years). All of them are right-handed and native speakers of Chinese with normal or corrected-to-normal vision. Signed informed consent was obtained from each participant prior to the experiment. The Medical Ethical Committee of the author’s university approved the experiment as compliant with the Declaration of Helsinki.

### Design

The experiment had a 2 (format familiarity: reading from left to right, reading from right to left) × 2 (word frequency: high frequency, low frequency) within-subjects design. Four blocks were constructed, each containing 96 sentences. There were 24 sentences in each condition, and the conditions were rotated across files according to a Latin square. Sentences in each condition were presented in a blocked format, and the order of the sentences in each block was random. Twelve practice sentences, three for each condition, were included at the beginning of each experimental block. In addition, there were 24 filler sentences (six in each condition) that appeared randomly throughout the block. After each of the 22 sentences, a yes/no comprehension question was presented. In total, each participant read 132 sentences. It generally took 15-20 minutes for each participant to complete this task.

### Materials

Drawing on a published lexicon database developed by Cai and Brysbaert (5), we selected 48 pairs of two-character words as target words. The unit of word frequency and character frequency was the occurrences per million words (OPM). Each word pair included an HF word and an LF word. The frequency difference between HF and LF words was reliably different. Numbers of strokes were matched for the HF and LF conditions. There was a marginally significant difference in first-character frequency between the HF and LF conditions. Table 1 shows the means and standard deviations.

We next constructed 96 Chinese sentences containing the target words but not including them at the beginning or end of the sentence. Each sentence was presented from left to right and from right to left. Sentences were between 16 and 23 characters in length (M = 19.40 characters, SD = 1.33). The experiment included four conditions: HF–left to right, HF– right to left, LF–left to right, LF–right to left (see Table 2). The experimental sentences were rated for naturalness, meaning the extent to which they are fluent, easy to understand, and compliant with norms (3). Ratings were given on a 7-point scale by 40 individuals who did not participate in the eye-tracking experiment. The predictability test required another 28 participants to provide the following words given the sentence context before the target words. Naturalness and predictability were matched for HF and LF, *ts* < 1.

**Table 1.** The statistical characteristics of the experimental sentences and target words

Target word	HF	LF
Word frequency	368.11 (470.72)	8.10 (7.56)
First- character strokes	7.52 (2.90)	8.15 (3.09)
Second- character strokes	7.65 (2.68)	7.42 (2.62)
Total strokes	15.50 (3.74)	15.23 (3.28)
First- character frequency	2481.58 (4724.37)	1037.85 (2807.45)
Second-character frequency	2699.36 (3623.92)	1206.97 (1809.50)
Naturalness	6.49 (0.39)	6.41 (0.38)
Predictability	0.15 (1.46)	0.10 (1.02)

Note. Standard deviations are provided in parentheses.

**Table 2.** Example Chinese stimuli from the four experimental conditions.

Target words	The familiarity of format	Sentence
High frequency	familiar format	医疗保险业***必须***遵循职业道德规范。
	unfamiliar format	。范规德道业职循遵***须必***业险保疗医
Low frequency	familiar format	医疗保险业***务必***遵循职业道德规范。
	unfamiliar format	。范规德道业职循遵***必务***业险保疗医

Note: Under the high frequency - familiar format, the high-frequency target words were presented from left to right. Under the high frequency - unfamiliar format, the high-frequency target words were presented from right to left. In the low frequency - familiar format, the low-frequency target words were presented from left to right; and in the low frequency - unfamiliar format, the low-frequency target words were presented from right to left. The English translation for example sentence is "The medical insurance industry must follow the professional ethics specification" under four conditions.

### Apparatus

The experiment recorded right-eye movements using Eyelink 1000 (SR Research, Canada). The sampling rate was 1000Hz, while the accuracy rate was a 0.5° visual angle. Stimuli were presented on a 19-inch Dell monitor at a resolution of 1024 × 768. Participants maintained a distance of 70 cm from the screen. Each character was 25 × 25 pixels, the visual angle was 0.80°, and the Song font was used.

### Procedure

Before the experiment, the participants were told to read sentences from right to left in different conditions. Participants needed to understand the meaning of sentences as quickly as possible and then to press the space bar to read the next sentence. For some sentences, a comprehension question followed, which participants had to answer as correctly as possible. Chin rests were used to ensure that the participants’ heads remained in a resting position with no head movement. Calibration was completed before the experiment to calculate the fixation point. Participants started the test after successful calibration. When necessary, eye location was recalibrated during the experiment. The experiment lasted about 20 minutes. Participants’ correctly answered 91.0% of the comprehension questions, indicating that the sentences were predominantly read and understood.

### Data preparation and analysis

In line with criteria from earlier research (3; 11; 12; 27; 30; 35; 36; 37), fixation durations shorter than 80 ms or longer than 800 ms were excluded. We also excluded data if: (1) a participant pressed the key incorrectly during the experiment, resulting in an interruption; (2) data were accidentally lost (e.g., due to head movement); (3) there were fewer than four gazes; or (4) data were outside three standard deviations. After excluding invalid data (2.8% of total data), we conducted data analysis.

We computed eight eye-movement measures for the target words: (1) first fixation duration (FFD)—the duration of the first fixation on a word, irrespective of the number of fixations; (2) Single fixation duration (SFD)—the fixation duration when only one fixation was made on the word during first-pass reading; (3) gaze duration (GD)—the sum of all fixations on a word before moving to another word; (4) regression-path duration (RPD)—the sum of all gaze times looking back to the current word; (5) Total time (TT)—the sum of all fixations on the target word, including regressions; (6) Skipping probability (SP)—the probability of skipping the target region in the first reading; (7) Refixation rate (RR)—the probability of the target region being gazed at multiple times in the first reading; (8) Average Initial landing position (ALP)—the distance to the beginning of the target word for the first time. The time index units (FFD, SFD, GD, RPD, and TT) were measured in milliseconds.

Collected data were analyzed using a linear mixed model (LMM) in the R environment (R Core Team, 2016), using the Ime4 package (4). We classify data as significant if the *t*-value exceeds 1.96 at the 5% level. Participants and items were specified as cross-random effects. We used posterior distributions for model parameters employing Markov-Chain Monte Carlo sampling to estimate *p*-values. Significance values reflected both subject and item variability (1). Log-transformed analysis was performed on the analysis indexes, and logistic mixed model Ime4 was performed on the skipping probability and refixation rate. The LMM was used to analyze word frequency, format familiarity, and their interaction as fixed factors. If there was a significant interaction between word frequency and format familiarity, the HF condition was compared with the LF condition from left to right (Comparison 1), and the HF condition and the LF condition were compared from right to left (Comparison 2).

## Experiment 1 Results

Table 3 shows the means and standard deviations of the eye movement measures for the target words. Table 4 shows the fixed effect estimates for FFD, SFD, GD, RPD, TT, SP, RR, and ALP for the target words.

**Table 3.** Eye-movement measures for the target words.

	Familiar format		Unfamiliar format	
	(reading from left to right)		(reading from right to left)	
	HF	LF	HF	LF
FFD (ms)	243(75)	266(88)	277(98)	281(101)
SFD (ms)	243(74)	267(88)	273(94)	279(102)
GD (ms)	275(111)	326(145)	389(189)	426(207)
RPD (ms)	331(199)	411(263)	532(363)	631(403)
TT (ms)	369(183)	483 (256)	619(334)	752(396)
SP	0.23(0.38)	0.18(0.32)	0.17(0.29)	0.11(0.26)
RR	0.12(0.25)	0.22(0.37)	0.34(0.44)	0.43(0.47)
AIP	0.91(0.55)	0.87(0.53)	0.80(0.51)	0.73(0.46)

Note: Standard deviations are provided in parentheses. FFD = First fixation duration; SFD = Single fixation duration; GD = Gaze duration; RPD = Regression-path duration; TT = Total time; SP = Skipping probability; RR = Refixation rate; AIP=Average initial landing position.

**Table 4.** Fixed effect estimates for FFD, SFD, GD, RPD, TT, SP, RR, and ALP for the target words.

	FFD	SFD	GD	RPD	TT	SP	RR	AIP
Intercept	5.521^***^	5.518^***^	5.752^***^	4.951^***^	6.127^***^	0.906^***^	-1.087^***^	-0.547^***^
Frequency	0.048^**^	0.056^**^	0.134^***^	0.217^*^	0.236^***^	-0.390^***^	0.565^***^	-0.077^*^
Familiarity	0.083^***^	0.063^***^	0.275^***^	0.478^***^	0.471^***^	0.534^***^	1.222^***^	-0.185^***^
Interaction	-0.070^*^	-0.071^§^	-0.058	-0.010	-0.030	0.026	-0.357^*^	-0.022
Compare 1	0.081^***^	0.092^***^	0.160^***^	0.205^**^	0.236^***^	-0.403^**^	0.085^***^	-0.079
Compare 2	0.014	0.018	0.105^*^	0.197^**^	0.236^***^	-0.376^**^	0.102^***^	-0.101^§^

Note: *** *p*<0.001, ** *p*<0.01, * *p*<0.05, ^§^ *p* < 0.1. FFD = First fixation duration; SFD = Single fixation duration; GD = Gaze duration; RPD = Regression-path duration; TT = Total time; SP = Skipping probability; RR = Refixation rate; IIP = Initial landing position. Interaction = the interaction between word frequency and the familiarity of format. Comparison 1: the high-frequency condition would be compared with the low-frequency condition in the familiar format (reading from left to right). Compare 2: the high-frequency condition and the low-frequency condition would be compared in the unfamiliar format (reading from right to left).

The results show that the format familiarity effect and word-frequency effect in the fixation duration (FFD were shorter in the HF than in the LF: *b* = 0.048, *SE* = 0.015, *t* = 3.213, *p* = 0.002. FFD was also shorter from left to right than from right to left: *b* = 0.083, *SE* = 0.015, *t* = 5.478, *p* < 0.001. SFD was shorter in the HF than in the LF: *b* = 0.056, *SE* = 0.018, *t* = 3.116, *p* < 0.01. SFD was also shorter from left to right than from right to left: *b*= 0.063, *SE*= 0.018, *t*= 3.434, *p*< 0.001. GD was shorter in the HF than in the LF: *b* = 0.134, *SE* = 0.023, *t* = 5.861, *p* < 0.001. GD was shorter from left to right than from right to left: *b* = 0.275, *SE* = 0.023, *t* = 11.971, *p* < 0.001. RPD was shorter in the HF than in the LF conditions: *b* = 0.217, *SE* = 0.096, *t* = 2.262, *p =* 0.025. RPD was also shorter from left to right than from right to left: *b* = 0.478, *SE* = 0.096, *t* = 4.995**,** *p* < 0.001. TT was shorter in the HF than in the LF condition: *b* = 0.236, *SE* = 0.046, *t* = 5.184, *p* < 0.001. TT was also shorter from left to right than from right to left: *b* = 0.471, *SE* = 0.032, *t* = 14.729, *p* < 0.001). The HF target words were skipped more often than the LF target word (*b* = -0.390, *SE* = 0.107, *t* = -3.657**,** *p* < 0.001). There was also skipped more often from left to right than from right to left (*b*= 0.534, *SE*=0.093, *t*= 5.736, *p*< 0.001). The refixation probability was higher in the LF than in the HF condition (*b*= 0.565, *SE*= 0.109, *t*= 5.175, *p*< 0.001). RR was also higher from left to right than from right to left (*b*= 1.222, *SE*= 0.090, *t*= 13.537, *p*< 0.001). Compared with right to left and LF words, there were shorter fixation durations, higher SP, and lower refixation probability for left to right and HF words, which is consistent with expectations and previous research (7; 15; 12; 19; 21; 22; 29; 37).

The center of the word is the best fixation position for eye movements, and the reading efficiency is highest at the best fixation position. The farther the fixation position is from the word center, the lower the fixation efficiency (2; 15; 39; 42; 23). Compared with the familiar format, the average initial landing position was closer to the beginning of the word when reading efficiency was lower from left to right (*b* = −0.19, *SE* = 0.04, *t* = −4.69, *p* < 0.001). This interference may be caused by reading cost from right to left (15; 21). There was significant word-frequency effect on ALP (*b* = −0.077, *SE* = 0.03, *t* = −2.43, *p* = 0.015).

Interestingly, the interaction between different indexes was not consistent. FFD and SFD generally are usually considered early indicators (21). In the early indexes, the interaction between format familiarity and word frequency was significant. The interaction was significant in the FFD (*b* = -0.070*, SE* = 0.030, *t* = -2.326, *p* = 0.021). From left to right, there was a significant difference between HF and LF (*b* = 0.048, *SE* = 0.015, *t* = 3.213, *p* = 0.002). From right to left, there was no significant difference between HF and LF (*b* = 0.014, *SE* = 0.022, *t* = 0.607, *p* = 0.545). There was a marginally significant interaction on the SFD (*b* = -0.071, *SE* = 0.036, *t* = -1.950, *p* = 0.053). The SFD was considered a good indicator of the semantic stage in vocabulary recognition and was greatly influenced by word frequency. There were significant differences between HF and LF from left to right (*b* = 0.092, *SE*= 0.025, *t*= 0.025, *p* < 0.001). However, there was no significant difference between HF and LF from right to left (*b*= 0.018, *SE*= 0.027, *t*= 0.656, *p*= 0.513). This finding suggests that format familiarity could modulate the word-frequency effect. Although there was no significant interaction in GD (*b* = -0.058, *SE* = 0.046, *t* = -1.270, *p*= 0.206), the word-frequency effect was smaller from right to left (*b* = 0.105, *SE* = 0.042, *t* = 2.504, *p=* 0.013) than from left to right (*b* = 0.160, *SE* = 0.043, *t* = 3.747, *p*< 0.001). However, the interaction was not significant in the other time indexes. The interaction was not significant in the RPD (*b* = -0.010, *SE* = 0.060, *t* = -0.164, *p* = 0.870). The differences between HF and LF were also significant from left to right (*b* = 0.205, *SE* = 0.063, *t* = 3.270, *p* < 0.005) and from right to left (*b* = 0.197, *SE* = 0.062, *t* = 3.181, *p* < 0.005). For the TT, the interaction was not significant (*b* = -0.030, *SE* = 0.067, *t* = -0.454, *p* = 0.650). The differences between HF than LF were significant from left to right (*b* = 0.235, *SE* = 0.048, *t* = 4.930, *p* < 0.001) and from right to left (*b* = 0.205, *SE* = 0.047, *t* = 4.357, *p* < 0.001). The interaction was not significant on the SP (*b* = 0.026, *SE* = 0.185, *t* = 0.142, *p* = 0.887). The differences between HF and LF were significant from left to right (*b* = -0.403, *SE* = 0.150, *t* = -2.681, *p =* 0.007) and from right to left (*b* = -0.378, *SE* = 0.131, *t* = -2.870, *p =* 0.004). The interaction was not significant for the average landing position (*b* = -0.022, *SE* = 0.078, *t* = -0.275, *p* = 0.783).This results was consistent with the previous researchers (29; 15; 21).

## Experiment 1 Discussion

Experiment 1 investigated the individual and interaction effects of word frequency and format familiarity on Chinese vocabulary recognition. In all eye-movement indexes, there were word-frequency effects and format familiarity effects, with best reading performance found in the left to right, HF condition. FFD and SFD represented the early indexes which reflected the early processing in Chinese vocabulary recognition (e.g., 28; 15; 21; 9). There was significant interaction effect on FFD: the word-frequency effect appeared from left to right but not from right to left. We also found a marginally significant interaction effect on SFD: the word-frequency effect appeared from left to right but not from right to left. FFD and SFD represent early processing. By contrast, we found no significant interaction effects on the other time indexes, indicating that format familiarity affects only early processing in word recognition. Thus, for the early indicators, a lack of right-to-left reading experience had a more significant influence on HF words. This could explain some results in previous studies (e.g., 21). There was also a significant interaction effect on RR, a sensitive indicator reflecting cognitive processing efficiency during fixation (e.g., 32; 3). Readers could fixate the optimal viewing position in fewer counts, thereby obtaining more information. This result suggests that format familiarity influences processing efficiency in word recognition.

The result in experment 1 may explain delay effect in previous study (21). The familiarity verification delays the appearing of word-frequency effect from right to left, the vocabulary processing was faster in the familiar format, the word-frequency effect only appeared on early indexes. According to the results of Experiment 1, the facilitation of format familiarity are due to features of the reading experience. The directional oculomotor activities in association with reading behind format familiarity may affect vocabulary recognition from top to bottom (7). There is rich experience from left to right, which caused the faster neural processing. While there is lack experience from right to left, which may have a delay in neural processing and reflect in the vocabulary recognition. Therefore, reading experience is considered as particularly important, reading training could improve reading experience quickly (e.g. 3; 24; 7). Based on the results of Experiment 1, we can infer that the impact of format familiarity on the word-frequency effect (the interaction effect of word frequency and format familiarity on the early indexes) shrinks or even disappears as related reading experience increases. This assumption is tested in Experiment 2.

## Experiment 2 Methods

In Experiment 2, participants first received 10 days of training in right-to-left reading, then completed reading tasks in which their eye movements were tracked. The training was intended to increase participants’ reverse-reading experience. Experiment 2 specifically investigated the roles of reading training and word frequency in Chinese vocabulary recognition, testing for changes in the word-frequency effect. To minimize the impact of confounding variables, Experiment 2 used the same participants as Experiment 1 and matched materials.

### Participants

All 40 undergraduate students began the 10-day reading training, but only 32 participants participated in the eye-movement tests—the other eight dropped out during training. Based on the Declaration of Helsinki, the Medical Ethical Committee of the author’s university approved the experiment.

### Design

Experiment 2 used a single-factor (word frequency: HF, LF) within-subjects design. Four block files were constructed, each containing 48 sentences. There were 24 sentences in each condition, and the conditions were rotated across files according to a Latin square. Sentences in each condition were presented in a blocked format, and the order of the sentences in each block was random. Six practice sentences, three for each condition, were included at the beginning of each experimental file. In addition, there were 12 filler sentences (six for each condition) that appeared randomly throughout the block. After every 11 sentences, a yes/no comprehension question was presented. In total, each participant read 66 sentences, all right to left.

### Materials

We used 24 pairs of two-character words as target words, which were different with that in Experiment 1. Based on a published lexicon database developed by Cai and Brysbaert (5), the OPM was the unit of word and character frequency. Each pair of words included an HF word and an LF word. The frequency difference between HF and LF words was reliably different. The numbers of strokes were matched for the HF and LF conditions. There were significant differences between the first character frequency of the HF and that of LF words. The second character frequency of the HF words was higher than that of the LF words. Table 5 shows the means and standard deviations.

Second, 48 Chinese sentences were constructed. The target words were within the sentences, not at the beginning or at the end of the paragrah. The experiments had two conditions in the experiment: HF– right to left, LF– right to left (see Table 6). The experimental sentences were rated for naturalness on a 7-point scale by 40 individuals who did not participated in the eye-tracking experiment. They were also rated for predictability by another 28 non-participating individuals.. Naturalness and predictability were matched for the HF and LF conditions.

The materials used in the reading training were 60 Chinese essays (average number of words M = 936, SD = 45) chosen from Chinese high school textbooks, all were reversed from left-to-right format to right-to-left format using reversing software (see the Appendix for samples of the reading materials).

**Table 5.** Statistical characteristics of the experimental sentences and target words

Target word	HF(24)	LF(24)
Word frequency	465.00(436.55)	6.24(5.63)
First- character strokes	8.04(2.37)	8.46(3.45)
Second- character strokes	7.33(2.78)	6.63(2.84)
Total strokes	15.38(3.75)	15.08(4.49)
First- character frequency	1501.60(1962.25)	494.67(599.69)
Second-character frequency	2955.16(3403.15)	1133.15(1745.39)
Naturalness	6.33(0.40)	6.36(0.42)
Predictability	0.04(0.20)	0.13(0.34)

Note: Standard deviations are provided in parentheses.

**Table 6.** Example Chinese stimuli from the two experimental conditions.

Target words	Sentence
High frequency	。师老语英的责负观乐*要需*校学际国
Low frequency	。师老语英的责负观乐*需急*校学际国

Note: The meaning of the Chinese sentence is that international schools need optimistic and responsible English teachers.

### Apparatus

The apparatus was the same as in Experiment 1

### Procedure

There were two stages: reading training (collective learning) and eye movement tracking (individual tests). For training, participants came to the laboratory every day. They sat in their allocated seats, each with a copy of the book containing the articles to be read was presented. Before reading began, the researcher gave the following instructions:

You will now read some articles. The sentences in the articles will be presented from right to left. Please read carefully word by word and understand the article as much as possible. Seven reading comprehension questions will appear after each article. You are required to select the most appropriate answer based on the article and fill in the answer.

Participants then started to read after understanding the instruction. After reading each article, they completed the reading comprehension questions before moving on to the following article. The entire reading experiment lasted for 30 minutes. Each participant completed one reading exercise per day, comprising five articles. After 10 days of reading training, eye-movement testing began using the same procedure as in Experiment 1. Participants correctly answered 93.0% of the comprehension questions during eye-movement testing, indicating that the sentences were predominantly read and understood.

### Data preparation and analysis

We applied the same data-selection criteria, analysis models, eye-movement measures, significance threshold, and p-value estimation approach as in Experiment 1 (e.g., 3; 12; 16; 30; 28; 37). After excluding invalid data (1.65% of the total data) according standards, which was mentioned in the experiment 1, data analysis was conducted. The LMM in this experiment analyzed the word frequency, the training effect, the format familiarity, the interaction of the word frequency and training, and the interaction of the word frequency and format familiarity as fixed factors. If there was a significant interaction between word frequency and format familiarity, the HF condition was compared with the LF condition from left to right before training (Comparison 1), and the HF condition and the LF condition were compared from right to left before training (Comparison 2). If there was a significant interaction between word frequency and training, the HF condition was compared with the LF condition from right to left before training (Comparison 2), and the HF condition and the LF condition was compared from right to left after training (Comparison 3).

## Experiment 2 Results

Table 7 shows the means and standard deviations of eye movement measures for the target words combined alongside the results of Experiment 1 for comparison. Table 8 shows the fixed effect estimates for FFD, SFD, GD, RPD, TT, SP, RR, and ALP for the target words.

**Table 7.** Eye movement measures for the target words

	Word frequency	Before reading training	Before reading training	After reading training
		From left to right	From right to left	From right to left
FFD	High	242(73)	276(97)	278(80)
	Low	266(88)	280(100)	282(86)
SFD	High	241(73)	272(93)	275(78)
	Low	264(86)	276(100)	280(84)
GD	High	274(109)	387(184)	348(157)
	Low	327(146)	427(208)	395(190)
RPD	High	332(199)	532(363)	448(323)
	Low	411(263)	631(403)	541(360)
TT	High	369(183)	616(333)	471(281)
	Low	484(257)	742(385)	609(339)
Skip	High	0.23(0.38)	0.17(0.29)	0.12(0.26)
	Low	0.18(0.32)	0.11(0.26)	0.12(0.22)
RR	High	0.12(0.26)	0.34(0.43)	0.24(0.38)
	Low	0.22(0.36)	0.43(0.49)	0.34(0.42)
AIP	High	0.91(0.56)	0.80(0.50)	0.83(0.53)
	Low	0.87(0.51)	0.73(0.44)	0.81(0.50)

Note: Standard deviations are provided in parentheses. FFD = First fixation duration; SFD = Single fixation duration; GD = Gaze duration; RPD = Regression-path duration; TT = Total time; SP = Skipping probability; RR = Re-fixation rate; AIP=average landing position.

**Table 8.** Fixed effect estimates for FFD, SFD, GD, RPD, TT, SP, RR, AIP for the target words

	FFD	SFD	GD	RPD	TT	SP	RR	AIP
Intercept	5.541^***^	5.531^***^	5.764^***^	5.967^***^	6.114^***^	0.153^***^	0.291^***^	-0.547^***^
Frequency	0.038^**^	0.035^*^	0.119^***^	0.180^***^	0.232^***^	-0.035^***^	0.096^***^	-0.077^*^
Format familiarity	0.078^***^	0.053^**^	0.253^***^	0.393^***^	0.431^***^	-0.070^***^	0.220^***^	-0.185^***^
Training	0.027^§^	0.027	-0.068^**^	-0.165^***^	-0.229^***^	-0.017	-0.103^***^	0.065
Interaction 1	-0.070^*^	-0.007^*^	-0.066	-0.037	-0.030	0.013	-0.017	-0.022
Interaction 2	0.004	-0.011	0.023	0.022	0.050	0.039^§^	0.017	0.048
Compare 1	0.084^***^	0.088^***^	0.156^***^	0.197^***^	0.235^***^	0.000^***^	0.000^***^	-0.079
Compare 2	0.014	0.015	0.090^**^	0.160^***^	0.205^***^	0.000^*^	0.000^***^	-0.101^§^
Compare 3	0.017	0.003	0.113^***^	0.182^***^	0.255^***^	0.000	0.000^***^	-0.052

Note: *** *p*<0.001, ** *p*<0.01, * *p*<0.05, ^§^ *p* < 0.1. Interaction 1 represented the interaction between word frequency and format direction. Interaction 2 represented the interaction between word frequency and training. The high-frequency would be compared with the low-frequency from left to right before training (Comparison 1). The high-frequency and the low-frequency would be compared from right to left before training (Comparison 2). The high-frequency and the low-frequency would be compared from right to left after training (Comparison 3).

In Table 8, the Comparison 3 results reveal no significant word-frequency effect on early fixation duration (FFD: *b* = 0.017, *SE* = 0.020, *t* = 0.875, *p* = 0.382. SFD: *b* = 0.003, *SE* = 0.027, *t* = 0.117, *p* = 0.907). However, there was a significant word-frequency effect on GD, LF, and RPD (Gaze duration was shorter in the HF than LF condition: *b* =0.113, *SE* = 0.031, *t* = 3.595, *p* < 0.001. Total time was shorter in the HF than LF condition: *b* = 0.255, *SE* = 0.047, *t* = 5.436, *p* < 0.001. RPD was shorter in the HF than LF condition: *b* = 0.182, *SE* = 0.043, *t* = 4.203, *p* < 0.001). In addition, RR was higher in the LF than in the HF condition (*b* = 0.000, *SE* = 0.000, *t* = 4.251, *p*< 0.001). Finally, we found no significant word-frequency effect on SP or ALP (skipping probability: *b* = 0.000, *SE* = 0.000, *t* = -0.271, *p* = 0.787. average landing position: *b* = -0.052, *SE* = 0.054, *t* = -0.963, *p* = 0.335).

The findings reveal a training effect after 10 days of reading training, which was similar with the previous study (7). In the early-stage indexes, there was a marginally significant training effect on FFD for HF and LF words (*b* = 0.027, *SE* = 0.014, *t* = 1.899, *p* = 0.059), which was shorter after training, but no significant training effect on SFD (*b* = 0.027, *SE* = 0.019, *t* = 1.452, *p* = 0.147). For all the other time indexes except SP, there was a significant training effect, with shorter duration after training (GD: *b* = -0.068, *SE* = 0.022, *t* = -3.044, *p* = 0.003; RPD: *b* = -0.165, *SE* = 0.031, *t* = -5.373, *p* < 0.001; TT: *b* = -0.23, *SE* = 0.033, *t* = -6.878, *p* < 0.001), more frequent skipping (RR: *b* = -0.103, *SE* = 0.015, *t* = -6.74, *p* < 0.001) and fixation point closer to the word center after training (AIP :*b* = 0.065, *SE* = 0.038, *t* = 1.691, *p* = 0.091) , apart from SP occurrences (*b* = -0.017, *SE* = 0.011, *t* = -1.51, *p* = 0.130).

Interaction 2 in Table 8 shows the interactions between word frequency and training. The interaction effects were not significant for all but one measure. Specifically, there was no significant differences between pre- and post-training values for FFD (interaction: *b* = 0.004*, SE* = 0.028, *t* = 0.130, *p* = 0.896), SFD (interaction: *b* = -0.011, *SE* = 0.037, *t* = -0.309, *p* = 0.758), GD (interaction: *b* = 0.023, *SE* = 0.044, *t* = 0.521, *p* = 0.603), RPD (interaction: *b* = 0.022, *SE* = 0.061, *t* = 0.353, *p* = 0.724), TT (interaction: *b*= 0.050, *SE*= 0.066, *t*= 0.758, *p*= 0.449), RR (interaction: *b* = 0.017, *SE* = 0.030, *t* = 0.548, *p* = 0.584) and average initial landing position (interaction: *b* = 0.048, *SE* = 0.077, *t* = 0.631, *p* = 0.528).We found a marginally significant interaction effect on SP (*b*= 0.039, *SE*= 0.022, *t*= 1.724, *p*= 0.085). The differences between HF and LF were significant before training (*b*= 0.000, *SE*= 0.000, *t*=-2.562, *p=* 0.011) but not significant after training (*b*= 0.000, *SE*= 0.000, *t*= -0.271, *p=* 0.787).

Table 8 combines the results of Experiment 2. There were significant word-frequency effects in FFD (*b* = 0.038, *SE* = 0.012, *t* = 3.294, *p* < 0.001), SFD (*b* = 0.035, *SE* = 0.015, *t* = 2.424, *p* = 0.016), GD (*b* = 0.119, *SE* = 0.018, *t* = 6.540, *p* < 0.001), RPD (*b* = 0.180, *SE* = 0.025, *t* = 7.134, *p* < 0.001), TT (*b* = 0.23, *SE* = 0.027, *t* = 8.500, *p* < 0.001), SP (*b =* -0.035, *SE* = 0.011, *t* = -3.191, *p* = 0.002), RR (*b* = 0.096, *SE* = 0.016, *t* = 5.856, *p* < 0.001) and ALP(*b* = -0.077, *SE* =0.032, *t* = -2.43, *p* = 0.015). In addition, there were significant format familiarity effects in all indexes (FFD: *b* = 0.078, *SE* = 0.015, *t* = 5.388, *p* < 0.001, SFD: *b* = 0.053, *SE* = 0.017, *t* = 3.139, *p* < 0.005, GD: *b* = 0.253, *SE* = 0.023, *t* = 11.206, *p* < 0.001. RPD: *b* = 0.393, *SE* = 0.031, *t* = 12.655, *p* < 0.001. TT: *b* = 0.431, *SE* = 0.034, *t* = 12.838, *p* < 0.001. SP: *b =* 0.000, *SE* = 0.000, *t* = -3.327, *p* = 0.001. RR: *b* = 0.220, *SE* = 0.016, *t* = 14.006, *p* < 0.001. ALP: *b* = -0.185, *SE* = 0.039, *t* = -4.692, *p* < 0.001).

The interaction effects between word frequency and format familiarity were similar to those found in Experiment 1. For the early indexes, the interaction effect was significant. We found a considerable interaction in FFD (Interaction: *b* = - 0.070*, SE* = 0.029, *t* = -2.442*,* *p* = 0.015. From left to right, there was a significant word-frequncy effect: *b* = 0.084, *SE* = 0.021, *t* = 4.055, *p* < 0.001. From right to left, there was no significant word-frequncy effect: *b* = 0.014, *SE* = 0.020, *t* = 0.694*,* *p* = 0.488) and SFD (Interaction: *b* = - 0.073*, SE* = 0.034, *t* = -2.155**,** *p* = 0.032. From left to right, there was a significant word-frequncy effect: *b* = 0.088, *SE* = 0.023, *t* = 3.879**,** *p*< 0.001. From right to left, there was no significant word-frequncy effect: *b* = 0.015, *SE* = 0.025, *t* = 0.584**,** *p* = 0.559). Although there was no significant interaction on GD (*b* = -0.066, *SE* = 0.045, *t* = -1.472**,** *p* = 0.142), the word-frequency effect was smaller from right to left (*b* = 0.090, *SE* = 0.031, *t* = 2.867**,** *p =* 0.004) than that from left to right (*b* = 0.156, *SE* = 0.032, *t* = 4.837, *p* < 0.001). We found no significant interaction effect on RPD (*b* = -0.037, *SE* = 0.062, *t* = -0.598, *p* = 0.550). There were significant word-frequncy effects from left to right (*b* = 0.197, *SE* = 0.044, *t* = 4.466, *p* < 0.001) and from right to left (*b* = 0.160, *SE* = 0.043, *t* = 3.683, *p* < 0.001). This finding suggests that format familiarity could impact the word-frequency effect.

The TT interaction was not significant (*b* = -0.030, *SE* = 0.067, *t* = -0.454, *p* = 0.650). The differences between HF and LF were significant from left to right (*b* = 0.235, *SE* = 0.048, *t* = 4.930, *p* < 0.001) and from right to left (*b* = 0.205, *SE* = 0.047, *t* = 4.357, *p* < 0.001). The interaction was not significant on SP (*b* = 0.013, *SE* = 0.022, *t* = 0.584, *p* = 0.559). There were significant word-frequency effects from left to right (*b* = 0.000, *SE* = 0.000, *t* = -3.327, *p* < 0.001) and from right to left (*b* = 0.000, *SE* = 0.000, *t* = -2.562, *p =* 0.011). The interaction was not significant on the RR (*b*= -0.017, *SE* = 0.031, *t* = -0.554, *p* = 0.580). There were significant word-frequncy effects from left to right (*b* = 0.000, *SE* = 0.000, *t* = 4.095, *p* < 0.001) and from right to left (*b* = 0.000, *SE* = 0.000, *t* = 3.539, *p* < 0.001). The interaction was not significant in the average landing position (*b* = -0.022, *SE* = 0.078, *t* = -0.275, *p* = 0.783). There were significant word-frequncy effects from left to right (*b* = -0.079, *SE* = 0.057, *t* = -1.395, *p =* 0.163) and from right to left (*b* = -0.101, *SE* = 0.054, *t* = -1.853, *p =* 0.064). These results are consistent with prior findings (e.g., 32; 15; 21).

## Experiment 2 Discussion

Experiment 2 investigated whether reading training could promote the word-frequency effect and whether there is a word-frequency effect in the early indexes (FFD and SFD) for right-to-left reading. We found significant training effects on all eye-tracking measures. However, the training effect was only marginally significant on FFD and non-significant on SFD. This inconsistency may result from the experience of reading right to left during word processing, which had a greater effect on late processing and a weaker effect on early processing.

There was no significant interaction between word frequency and training in Experiment 2, and only a marginally significant interaction effect on RR. These results show that reading training promoted HF condition and LF condition equally through reducing RR. In Experiment 1, we found a word-frequency effect from left to right for all indexes, but this effect was delayed from right to left and did not appear in the early indexes (FFD and SFD). The delayed effect was also observed by (21): under the unfamiliar condition without word-segmented clues, the appearance time of the word-frequency effect was delayed by 21ms. We found that right-to-left reading experience of the right to left format was improved through training, which was smiliar with previous study (7). However, there was no word-frequency effect in the early indexes (FFD and SFD). The other time indexes involved deeper semantic processing, and reading experience had a greater effect on semantic processing and vocabulary recognition from top to bottom. This confirms the hypothesis of Experiment 1 to some extent. The oculomotor nervous system processes familiar formats quickly, and reading training increases reading experience from right to left. However, this increase in reading experience impairs deep-level reading processing, such as semantics and later word recognition, but has little impact on early processing brain (for example, primary visual cortex). However, FFD and SFD are more affected by the visual characteristics of Chinese characters, which likely explains the non-significant improvement after training.

## General Discussion

This study manipulated format familiarity and word frequency to investigate whether the former modulates the latter in Chinese vocabulary recognition. We also examined whether right-to-left reading training influences the word-frequency effect. Through two experiments, we further examine the processing mechanism of word segmentation and vocabulary frequency, thereby revealing the delay effect of previous research (21).

Experiment 1 suggested that format familiarity influences lexical processing in Chinese, especially in the early stages. This maybe explain by the left-side bias. For Chinese characters, a left-side processing bias, in which observers rely more heavily on information conveyed by the left side of stimuli than the right side of stimuli, which was related with habitual format familiarity (34). Reduced left-side bias effects were observed among right-to-left readers as compared with left-to-right readers. The left-side bias is explained by visuospatial asymmetries, which may also be influenced by the directional oculomotor activities involved in reading and writing. Experiment 2 then explored whether reading training changed the word-frequency effect on eye-tracking indexes. While readers of Chinese lack right-to-left reading experience, the eye-tracking results show that training had a significant effect on SFD. However, there was no significant interaction effect between word frequency and training. These findings indicate that the early indexes may be more affected by the visual characteristics of Chinese characters.

Reading experience affects the speed and efficiency of Chinese vocabulary recognition. Ma (21) dismissed the visual familiarity hypothesis, proposing that the inter-word spaces lower reading time by reducing lateral masking and facilitating word segmentation. However, word segmentation and word recognition are unified and indistinguishable. There seems to be a trade-off between familiarity and word segmentation (7), suggesting that the influence of familiarity cannot be excluded. Our study manipulated format familiarity using reading direction; the difference in format familiarity was attributable to the difference in reading experience. In the familiar condition of left to right reading, Chinese readers had a higher skipping rate and lower refixation probability. Reading experience is a high-level cognitive factor with a top-down effect on vocabulary recognition. The richer the reading experience, the higher the reading performance and efficiency, and the closer the fixation point to the word center (3; 7; 13; 42). The results of Experiment 2 support the view that reading experience influences vocabulary recognition in Chinese reading. The word-frequency effect could reflect the vocabulary-recognition stage sensitively (19; 20; 21; 22; 32; 38). Notably, the interaction between format familiarity and word frequency was significant in the early indexes, which may indicate a delay in the word-frequency effect in right-to-left reading. This delay is similar to that found by Ma (21).

Based on the SWIFT model, word segmentation and word recognition are top-down and bottom-up unified processes (9; 14). When word segmentation is completed, vocabulary recognition is too. However, the delayed word-frequency effect from right to left suggests that word segmentation processing may be independent in Chinese reading, consistent with the E-Z Reader model (31; 33; 21; 7), which proposes that readers conduct word segmentation and then vocabulary recognition when reading. From left to right, participants fully or partially completed word segmentation in the preview stage, then entered the vocabulary-recognition stage. Therefore, the word-frequency effect appeared in the early-fixation stage. From right to left, however, participants did not have sufficient reading experience and thus paid a higher cost, with word segmentation spilling over from the preview stage into the familiarity-verifying stage, thereby delaying the word-frequency effect on vocabulary recognition. If word segmentation and vocabulary recognition are the same process, there should always be an interaction between format familiarity and word frequency. The results showed such an interaction in the early indexes (FFD and SFD); however, in the late indexes, the word-frequency effect was significant from left to right and from right to left, meaning that format familiarity had no impact on the word-frequency effect, that the word segmentation process had been completed, and that the vocabulary-recognition stage had begun. Therefore, the word-frequency effect always existed. In short, the processes of word segmentation and vocabulary recognition in late reading may be sequential, rather than concurrent. However, this processing mechanism is complicated and also involves character recognition. Whether there is an interaction between different levels should be explored in future research.

### Conclusion

We draw five main conclusions from the study’s results. First, format familiarity affects vocabulary recognition. From left to right, adult readers have shorter fixation times, higher skipping rates, and viewing positions closer to the word center. Second, word frequency affects vocabulary recognition in Chinese reading. Third, right-to-left reading training can improve reading performance. Fourth, the interaction effect of format familiarity and word frequency on the early indexes was significant: there was a word-frequency effect from left to right but not from right to left. Finally, we infer that word segmentation and vocabulary recognition may be sequential in Chinese reading.

### Ethics and Conflict of Interest

The author declares that the contents of the article are in agreement with the ethics described in http://biblio.unibe.ch/portale/elibrary/BOP/jemr/ethics.html and that there is no conflict of interest regarding the publication of this paper.
